# Investigating the Effect of Isoelectric Points on the Gas-Phase Stability of Native-like Proteins Analyzed in Positive- versus Negative-Ion Mode by IMS-MS

**DOI:** 10.1021/acs.analchem.5c04295

**Published:** 2026-02-04

**Authors:** Alexis N. Edwards, Madeline G. Bannon, Michael S. Cordes, Elyssia S. Gallagher

**Affiliations:** Department of Chemistry and Biochemistry, 14643 Baylor University, One Bear Place #97348, Waco, Texas 76798, United States

## Abstract

Native ion mobility spectrometry-mass spectrometry (IMS-MS) is routinely used for analysis of folded proteins and protein complexes. For many proteins, the three-dimensional structure is maintained during electrospray ionization (ESI) as the protein transitions to the gas phase, allowing for detailed investigation of the gaseous, ionic protein’s structure and stability. Much of the native IMS-MS research has been conducted in positive-ion mode (+ESI), even when the protein of interest has a net-negative charge in solution at physiological pH. We hypothesize that analyzing a protein in the polarity that is opposite to its solution-phase charge, such as analyzing net-negative proteins by +ESI-MS, disrupts the network of noncovalent-bonding interactions within the protein to a greater extent than using the polarity that matches the protein’s solution-phase charge, resulting in differences in protein stability. Herein, we show that while most protein ions have similar initial, folded structures in +ESI and negative-ion mode (−ESI), positive and negative ions exhibit significant differences in gas-phase stability. Furthermore, the energy required to cause this unfolding is often greater in the polarity corresponding to the solution-phase charge of the protein, indicating that the protein is more stable in that polarity. Thus, this work highlights the necessity of considering polarity when conducting native IMS-MS experiments.

## Graphical abstract

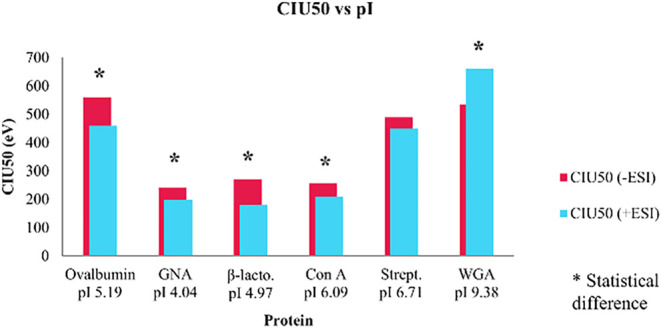

## Introduction

In native mass spectrometry (nMS), the native-like structures of proteins are maintained in the gas phase by preserving the secondary, tertiary, and quaternary structures.^1–7^ The preservation of native-like protein structures is primarily accomplished via the use of electrospray ionization (ESI), a soft ionization method that forms a distribution of multiply charged protein ions. These multiply charged ions can then be investigated to characterize stoichiometry, oligomeric composition, and ligand binding.^8–10^

During ESI, a potential is applied to the sample-containing solution. In an aqueous spray solvent, H_3_O^+^ and OH^–^ are produced in positive-ion mode (+ESI) and negative-ion mode (−ESI), respectively.^11^ The buildup of like-charges within the solution results in the formation of a Taylor Cone at the capillary tip, which ejects charged droplets that travel toward the inlet of the mass spectrometer. Droplets undergo evaporation and fission until complete analyte desolvation, leading to the release of folded, gas-phase protein ions. As the droplets shrink, H_3_O^+^ or OH^–^ can become concentrated, causing the pH of the droplet to decrease or increase, respectively. Shifts in pH can denature proteins. Therefore, to maintain native-like protein structures, nMS is conducted with solutions containing volatile salts, usually ammonium acetate, that provide ionic strength and minimize pH changes compared to spraying from pure water.^11,12^ Droplet pH in ESI is influenced by flow rate, solvent composition, and applied voltage,^13^ but often approaches values near the p*K*_a_ of acetate (4.76) or ammonium (9.25) in +ESI or −ESI,^12,14^ respectively. During ESI, changes in overall protein charge take place via proton transfer at chargeable amino acid residues, resulting in the formation of protonated or deprotonated protein ions with the charged sites being dependent on the relative proton affinities of ammonium, acetate, and chargeable amino acids as proteins near complete desolvation.^15–19^

Ion mobility spectrometry (IMS) measures an ion’s mobility through an inert gas under the influence of an electric field. Thus, mobility is directly influenced by the ion’s size, shape, and charge.^20^ A protein’s mobility can be used to estimate its structure by calculating an average momentum-transfer collisional cross section (CCS). In addition to structural characterization, IMS-MS can investigate the gas-phase stability of protein complexes when paired with collision-induced unfolding (CIU). Specifically, in CIU experiments, protein ions are accelerated into a cell filled with inert gas molecules and undergo multiple collisions with higher frequency and energy relative to IMS. This converts kinetic energy to internal, vibrational energy, disrupting noncovalent interactions and inducing unfolding of the protein ions.^21^ Unfolding alters the protein structure, which can then be quantified by monitoring the protein’s mobility during subsequent IMS-MS measurement.^22^ Prior work with mutant proteins has shown that proteins with similar intramolecular interactions have similar CIU fingerprints, while proteins with different intramolecular interactions have different CIU fingerprints.^23^ Therefore, gas-phase protein stability is correlated with intramolecular interactions.

Past native IMS-MS work has primarily been conducted in +ESI given that -ESI has the reputation of being unreliable for native-like protein analysis due to the occurrence of corona discharge,^24^ and charge-state distributions that may not be representative of folded proteins.^25^ In recent years, −ESI has been increasingly used in CIU and IMS-MS, with recent work demonstrating that −ESI-MS is capable of discerning denatured and native-like protein structures.^26,27^ Prior work showed that charge manipulation of folded proteins, such as charge reduction, to achieve similar absolute charge states in both ion modes, results in CCS values of protein cations that are comparable to their anion counterparts.^26,28^ This suggests that gas-phase structures are weakly dependent on protein charge and ionization polarity.^23,26,28^ Yet, Hong et al. showed that, in the absence of charge reduction, the gas-phase stability of two proteins (avidin and β-lactoglobulin) is sensitive to ionization polarity, highlighting that gas-phase stability is not consistent between +ESI and −ESI.^29^

Herein, we explore the relationship between the solution-phase charge of proteins and their stability as either positive or negative ions in the gas phase. We hypothesize that proteins with a net-negative charge in neutral solution experience greater changes to their intramolecular interactions during +ESI; therefore, becoming less stable in the gas phase when compared to −ESI. We hypothesize that the inverse is true for positively charged proteins analyzed via −ESI. To investigate the effect of ESI charging on the gas-phase structure and stability of intact proteins, 10 proteins with a range of oligomeric states and isoelectric points (pI), which define the pH at which proteins have no net charge in solution (see Table 1), were subjected to CIU-IMS-MS in both +ESI and −ESI. Here, we show that most proteins have comparable folded CCS in +ESI and −ESI, consistent with prior data.^26,28–30^ Though, several of these proteins unfold to extended structures that are not comparable in +ESI and −ESI. Additionally, we show that many proteins require significantly more energy to unfold in one polarity, which often matched their solution-phase charge at physiological pH. Particularly, proteins with a net-negative charge in solution are shown to be significantly more stable in −ESI than in +ESI. Furthermore, disulfide bridges stabilize proteins in both +ESI and −ESI. Therefore, our data indicates that a protein’s gas-phase stability may be inconsistent across MS polarities, highlighting that the polarity used to perform native IMS-MS experiments is an important consideration in experimental design.

**Table 1:** Analyzed Proteins and Structural Details

protein	number of subunits^a^	molecular weight (kDa)	disulfide bridges^b^	theoretical pI^c^	theoretical *z* at pH 7^c^	experimental pI^d^
ovalbumin	1	44.3	1	5.19	–10.1	4.5
*Galanthus Nivalis* agglutinin, GNA	2	23.9	2	4.04	–7.99	n/a
β-lactoglobulin	1	18.2	2	4.97	–5.89	4.8–5.2
α-lactalbumin	1	14.2	4	5.09	–5.49	4.2–4.6
concanavalin A, con A	1	25.6	0	6.09	–3.41	4.5–5.5, 6.6–7.1
streptavidin	4	55.0	0	6.71	–1.23	5.0–6.0
ubiquitin	1	8.56	0	7.02	+0.01	6.79
wheat germ agglutinin, WGA	2	34.4	32	9.38	+5.36	8.5
cytochrome *c*	1	12.3	0	11.30	+7.40	10.0–10.5
lysozyme	1	14.2	4	11.07	+8.36	11.4

a The number of subunits listed represents the analyzed oligomeric state, which does not always match the biological oligomeric state. However, these oligomers were selected because they consistently had overlapping charge states between ion modes.

b The number of disulfide bridges are based on UniProt records.

c PROPKA v3.5.0 ^31,32^ was used to determine the theoretical pI and *z* for the analyzed, folded proteins or oligomers at pH 7. See SI Table S1 for associated PDB files.

d Reported pI values from literature.^33–47^

## Materials and Methods

### Materials

Lyophilized proteins were from Sigma-Aldrich (St. Louis, MO). Methanol was from Thermo Fisher (Thermo Fisher Scientific Inc., Waltham, MA). All other materials were from VWR (Radnor, PA). Nanopure water was acquired from a Purelab Flex 3 purification system (Elga, Veolia Environment S.A., Paris, France).

### Sample Preparation

Folded proteins (see Tables 1 and S1) were prepared in 200 mM ammonium acetate (pH ∼ 7) and desalted using Micro Bio-Spin P-6 gel columns (Bio-Rad, Mississauga, ON). Concanavalin A (con A) was analyzed at 20 μM, while all other proteins were analyzed at 10 μM. Denatured myoglobin, cytochrome *c*, and ubiquitin (10 μM) were prepared for CCS calibration in 49:49:2 (v/v/v) methanol: water: formic acid for +ESI and in 49:49:2 (v/v/v) methanol: water: ammonium hydroxide for −ESI.

### Native IMS-MS Analysis

Glass capillaries were pulled using a P-1000 micropipette puller (Sutter Instrument Company, Novato, CA). Samples and calibrants were sprayed from pulled capillaries (inner diameter ∼1 μm) and analyzed with a Synapt G2-S High-Definition MS (Waters Corporation, Milford, MA). The trap and transfer cells contained Ar, the helium cell contained He, and the IMS cell contained N_2_. IMS wave velocity was 350 m/s, while IMS wave height was either 7 or 9 V, depending on the protein size (see Table S2). Proteins with larger mass required a wave height of 9 V to ensure that all the ions migrated out of the IMS cell prior to the next ion injection. Mass spectra were collected and signal averaged over 30 s. Additional instrument details can be found in the Supporting Information (Table S2).

To limit systematic error due to repositioning of the ESI tip between runs,^48^ the *XYZ*-stage position was optimized and kept consistent for all replicates. Approximately 5 μL of sample was loaded into a glass capillary, which was then mounted on the *XYZ*-stage. Electric potential was delivered to the sample by a platinum wire inserted into the capillary and placed in contact with the solution. The voltage applied to the tip holder ranged from 0.60 to 1.2 kV for native samples, and 0.80 to 1.40 kV for denatured samples. Nine replicates in each ion mode were collected for all proteins with measurements spread across 8 days over 3 weeks. These replicates were conducted in pairs by collecting data in both ion modes consecutively. To accomplish this, a CIU experiment was collected in +ESI before the instrument polarity was changed to −ESI. A second CIU experiment was performed, beginning with −ESI to counteract any changes in protein stability due to pH changes during ESI.^14^ This allowed for use of the same capillary, at the same position in relation to the instrument inlet, for each +ESI/–ESI replicate pair. A new tip was used for each +ESI/–ESI replicate pair. CIU experiments were accomplished by increasing collision voltage (CV) to the trap cell in 5 V steps, from 0 V to a maximum of 120 V.

### Data Analysis

Charge states for each protein were calculated using peaks with adjacent *m*/*z*. Following the recommendations of Gabelica et al.,^49^ experimental CCS values reported in this manuscript are labeled ^TW^CCS_N2→He_, where the superscript indicates the type of IMS used (traveling-wave, TW) and the subscript indicates that experimental values were measured using N_2_ and then converted to He with calibrant values. ^TW^CCS_N2→He_ values and their corresponding uncertainties were calculated using previously described protocols (see Table S3 for drift tube CCS (^DT^CCS_He_) and Figure S1 for calibration curves).^50,51^ Independent calibration curves were generated for both ion modes, as well as for both the 7 and 9 V IMS wave heights (totaling four calibration curves). For each protein, the arrival time at each CV was used to determine a corresponding ^TW^(CCS ± CCS_uncert_)_N2→He_. When the mean arrival time increased, resulting in a significant change to ^TW^CCS_N2→He_ (at the 95% confidence level), the resulting structure was considered a new feature. The change in CCS and its corresponding uncertainty (ΔCCS ± ΔCCS_uncert_) was calculated following eqs 1 and 2, respectively.

1 $$\Delta \mathrm{CCS}={\mathrm{CCS}}_{\mathrm{extended}}-{\mathrm{CCS}}_{\mathrm{folded}}$$

2 $$\Delta {\mathrm{CCS}}_{\mathrm{uncert}}=\sqrt{{{(\mathrm{CCS}}_{\mathrm{extended},\mathrm{uncert}})}^{2}+{\left({\mathrm{CCS}}_{\mathrm{folded},\mathrm{uncert}}\right)}^{2}}$$

Here, CCS_folded,uncert_ and CCS_extended,uncert_, are the propagated uncertainties for the CCS of the folded and extended structures, respectively.

For each protein replicate, CV values were converted to laboratory frame energy (eV) by multiplying CV by the absolute charge of the analyte. The corresponding arrival time was plotted versus eV, yielding CIU fingerprints. CIU fingerprints in this manuscript were generated by averaging all individual replicate CIU fingerprints (n was a minimum of eight replicates). All CIU fingerprints were made in CIUSuite2.^52^ The CV at which 50% of the species unfolded were determined by calculating CIU50 ^52^ values. CIU50 values for replicate analysis are presented as the average ± standard deviation. To calculate CIU50 values using CIUSuite2, the following parameters were used: minimum feature length (steps) = 2, feature allowed width (drift axis units) = 0.5, and maximum CV gap length (CV steps) = 1. Individual CIU50 values for each replicate analysis were determined to calculate an average CIU50 and a corresponding standard deviation (see Table S4). For some proteins, different values were chosen for the highest CV (*e*.*g*., 540 eV versus 480 eV) in +ESI and -ESI as they were the last CV step where the signal-to-noise ratio (S/N) was greater than 3. *F* tests and *t*-tests (95% confidence level) were used to determine if ^TW^CCS_N2→He_ and CIU50 were significantly different between ion modes.

### PROPKA

*PROPKA 3*.*5*.*0* ^31,32,53–55^ was used to determine protein pI and predict solution-phase charge at pH 7. Briefly, *PROPKA* predicts p*K*_a_ values of ionizable groups in a protein via a user-defined crystal structure (Table S1). These p*K*_a_ values are then used to determine the pI for the protein, from which a theoretical charge can be calculated at varying pH.

### Molecular Dynamics Simulations

Molecular dynamics (MD) simulations were used to determine the degree to which disulfide bridges stabilize gaseous, ionic protein structures after ESI. The GROMACS 2022.3 package^56^ was used to conduct the MD simulations with the CHARMM36 force field.^57^ To study the gas-phase stability of proteins with disulfide bridges, the model proteins lysozyme (PDB: 1DPX^58^) and α-lactalbumin (PDB: 1F6R^59^) were used. Additional details can be found in the Supporting Information.

## Results and Discussion

Ten proteins were chosen for analysis in +ESI and −ESI in this work (Table 1). These proteins can be broken into three groups depending on the net-charge in solution at pH 7: net-negative (with pI < 6.5), net-neutral (with 6.5 ≤ pI ≤ 7.5), and net-positive (with pI > 7.5). Five of the ten proteins have a net-negative charge at pH 7, though four of these proteins (con A, β-lactoglobulin, ovalbumin, and α-lactalbumin) are routinely analyzed via +ESI-IMS-MS.^18,28^ Additionally, each protein had charge-state distributions in +ESI and −ESI that resulted in at least one overlapping charge state (Figure 1), allowing for direct comparison of ^TW^CCS_N2→He_ and CIU50 in both ion modes.

**Figure 1:**
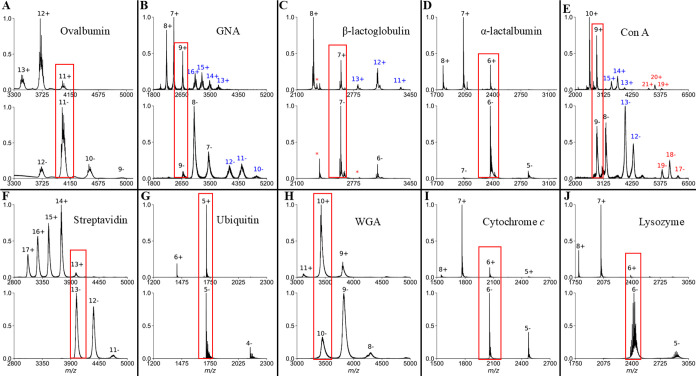
Representative mass spectra in +ESI (top) and −ESI (bottom) for (A) ovalbumin, (B) GNA, (C) β-lactoglobulin, (D) α-lactalbumin, (E) con A, (F) streptavidin, (G) ubiquitin, (H) WGA, (I) cytochrome *c*, and (J) lysozyme. Proteins that have blue or red charge states indicate additional oligomeric states that were detected. Outlines in red boxes are overlapping charge states that are compared herein. *Indicates contaminant peaks.

### Folded ^TW^CCS_N2→He_ are often Comparable for Proteins Ionized in Each Ion Mode

To compare folded structures in +ESI and −ESI, each protein was sprayed under native conditions and analyzed using instrumental parameters that limited unintentional ion activation. When comparing overlapping charge states, seven of the ten proteins (ovalbumin, β-lactoglobulin, α-lactalbumin, streptavidin, ubiquitin, cytochrome *c*, and lysozyme) exhibit similar ^TW^CCS_N2→He_ for the initial, folded structures (Tables 2 and S4). Two of the three net-positive proteins, both net-neutral proteins, and three of the five net-negative proteins follow this trend. Several papers have compared CCS values of folded proteins in +ESI and −ESI.^26,28,30,60^ Collectively, these papers concluded that gas-phase structures for proteins are comparable across ion modes when sprayed under native conditions. Observing comparable ^TW^CCS_N2→He_ from +ESI and −ESI suggests that, for these seven proteins, the gas-phase structures are not significantly different as either positive or negative ions, indicating that the noncovalent interactions associated with protein structure are similarly maintained during ESI in both ion modes.

**Table 2:** Comparison of Folded and Extended ^TW^(CCS ± CCS_uncert_)_N2→He_ for Proteins Analyzed in +ESI and −ESI

protein^a^	*z*	initial, folded ^TW^(CCS ± CCS_uncert_)_N2→He_ (Å^2^)	*n* ^b^	within uncertainty^c^	final, extended ^TW^(CCS ± CCS_uncert_)_N2→He_ (Å^2^)	*n* ^b^	within uncertainty^c^	ΔCCS (Å^2^)
ovalbumin	11–	3500 ± 110	9	√	4000 ± 130	9	√	600 ± 170
	11+	3400 ± 50	9		4040 ± 60	9		610 ± 80
GNA	9–	2180 ± 60	9		2540 ± 80	9		360 ± 100
	9+	2100 ± 30	9		2670 ± 40	9		560 ± 50
β-lactoglobulin	7–	1640 ± 40	9	√	1880 ± 50	9		240 ± 70
	7+	1640 ± 40	9		1960 ± 30	9		320 ± 20
α-lactalbumin	6–	1380 ± 40	9	√	1400 ± 40	8	√	20 ± 60
	6+	1410 ± 20	9		1410 ± 20	9		N/A^d^
concanavalin A	9–	2150 ± 70	9		2840 ± 70	9		470 ± 120
	9+	2090 ± 40	9		2880 ± 50	9		570 ± 70
streptavidin	13–	3900 ± 120	9	√	4700 ± 150	9		800 ± 200
	13+	3970 ± 60	9		4960 ± 70	9		1000 ± 90
ubiquitin	5–	960 ± 30	9	√	1020 ± 40	8		60 ± 40
	5+	990 ± 20	9		1070 ± 20	9		80 ± 20
WGA	10–	2680 ± 80	9		3070 ± 90	9	√	390 ± 120
	10+	2560 ± 50	9		2920 ± 40	9		370 ± 60
cytochrome *c*	6–	1240 ± 40	9	√	1570 ± 50	9		330 ± 60
	6+	1260 ± 20	9		1380 ± 20	9		130 ± 30
lysozyme	6–	1390 ± 40	9	√	1720 ± 60	9	√	330 ± 70
	6+	1420 ± 20	9		1710 ± 20	9		290 ± 30

a Proteins are listed in order of increasing theoretical charge at pH 7.

b n represents the number of replicates.

c √ indicates that the ^TW^CCS_N2→He_ were within measurement uncertainty at the 95% CI when comparing values for +ESI and −ESI.

d N/A in the ΔCCS column indicates that the species did not unfold prior to dissociation.

For the three proteins (GNA, con A, and WGA) that did not have comparable, folded CCS, ^TW^CCS_N2→He_ was more compact in +ESI compared to −ESI. At pH 7, GNA and con A have a net-negative charge, while WGA has a net-positive charge (Table 1). IMS-MS experiments and simulations have shown that amino-acid side chains at the protein surface can collapse onto the protein surface during desolvation,^61,62^ causing compaction of the protein upon entering the gas phase. We hypothesize that for these three proteins, gas-phase compaction may be more extensive for the positive-protein ions compared to the negative-protein ions. Because these three proteins are lectins, or carbohydrate-binding proteins, the carbohydrate-binding pocket may be influencing the observed structural difference in +ESI and −ESI mode. That is, the greater compaction in +ESI may correlate to the empty carbohydrate-binding pockets causing greater protein compaction in positive-protein ions compared to negative-protein ions.

### Extended ^TW^CCS_N2→He_ are Different in both Polarities for Many Proteins

We subjected proteins to CIU in both +ESI and -ESI to compare the ^TW^CCS_N2→He_ of the extended structures. After unfolding by CIU, four out of the ten proteins had extended ^TW^CCS_N2→He_ that are comparable within experimental uncertainty in +ESI and −ESI (Table 2), suggesting similar extents of unfolding. In solution, two of these proteins (ovalbumin and α-lactalbumin) are net-negative and two proteins (WGA and lysozyme) are net-positive.

GNA and con A, both net-negative proteins in solution, had folded structures that were significantly smaller in +ESI compared to −ESI. Unlike WGA, the third lectin in this study, both GNA and con A unfold to extended ^TW^CCS_N2→He_ that are different, with the expanded structure being significantly larger in +ESI compared to −ESI (Table 2). Because the +ESI structures go from being significantly smaller when folded, to significantly larger when extended, the ΔCCS in +ESI is much larger than the ΔCCS observed for −ESI. For example, the 9– ions for GNA have ΔCCS of (360 ± 100) Å^2^ while the 9+ ions have ΔCCS of (560 ± 50) Å^2^. This indicates that the extended structures in +ESI and −ESI are not the same. We hypothesize that for these two lectins, the cationic proteins achieve a higher degree of unfolding, which may also be associated with the carbohydrate-binding sites. In comparison, WGA unfolds to extended ^TW^CCS_N2→He_ that are within experimental uncertainty in +ESI and −ESI. Though WGA is also a lectin, this protein contains more disulfide bridges than GNA and con A (Table 1). Disulfide bridges will be examined in greater detail in the next section.

Cytochrome *c*, a protein with a net-positive charge in solution at pH 7, exhibited different extents of unfolding in +ESI and −ESI. The 6- ions experienced two unfolding events, while the 6+ ions experienced a single unfolding event; thus, the 6– ions had three distinct structures (initial folded, intermediate, and final extended), while the 6+ ions had two distinct structures (initial folded and final extended). The 6– ions had an extended ^TW^CCS_N2→He_ of (1570 ± 50) Å^2^ that was significantly larger than the extended ^TW^CCS_N2→He_ of the 6+ ions (1380 ± 20) Å^2^. When comparing the folded and extended structures, the 6– ions experience a ΔCCS of (330 ± 60) Å^2^ while the 6+ ions experience a smaller increase in CCS (ΔCCS) of (130 ± 30) Å^2^. The significant difference in ΔCCS is due to the 6– ions experiencing a second unfolding event compared to the 6+ ions (Table 2). Interestingly, the extended ^TW^CCS_N2→He_ (1380 ± 20) Å^2^ for the 6+ ion was comparable to the intermediate ^TW^CCS_N2→He_ (1390 ± 40) Å^2^ of the 6– ion. This suggests that the 6+ ions do not unfold to the fully extended structure exhibited by the 6- ions prior to loss of ion signal.

Proteins analyzed by CIU using both +ESI and −ESI exhibited diverse unfolding behaviors. Four of the ten proteins showed similar extended structures across both ionization modes, while the remaining six displayed significant differences. Two net-negative proteins, GNA and con A, had smaller folded structures in +ESI that unfolded to larger extended structures than in −ESI, resulting in greater ΔCCS values, thus suggesting more extensive unfolding for the cations. Cytochrome *c*, a net-positive protein, exhibited distinct unfolding pathways that suggest the cationic species underwent incomplete unfolding compared to the anion species. Overall, these findings indicate that ionization mode influences the extent of unfolding and the structural transitions observed in CIU experiments.

### Proteins with Disulfide Bridges are Stable in Both Polarities

For both lysozyme and α-lactalbumin, the average ^TW^CCS_N2→He_ were within experimental uncertainty in +ESI and −ESI for both the initial, folded structures and the final, extended structures (Table 2). However, the 6+ and 6- ions of lysozyme and α-lactalbumin showed a high resistance to unfolding with >90% of the ions retaining their folded ^TW^CCS_N2→He_ values even at high CV (Figure 2). Replicate data showed similar trends (Figure S2). While CCS values provide a description of gas-phase structures, CIU experiments provide information on the stability of the gas-phase protein structures. Furthermore, the number and pattern of disulfide bridges present in antibodies has been characterized by CIU.^63^ Lysozyme and α-lactalbumin both contain the glycosyl hydrolases family 22 (GH22) domain, which has four disulfide bridges that span ∼94% and ∼93% of the amino acid sequence, respectively (Figure S3). Thus, we hypothesize that rather than effects from protein pI and ionization polarity, the unfolding behavior of these proteins is controlled to a greater extent by the presence of stabilizing disulfide bridges that prevent routine unfolding during collisional activation.

**Figure 2:**
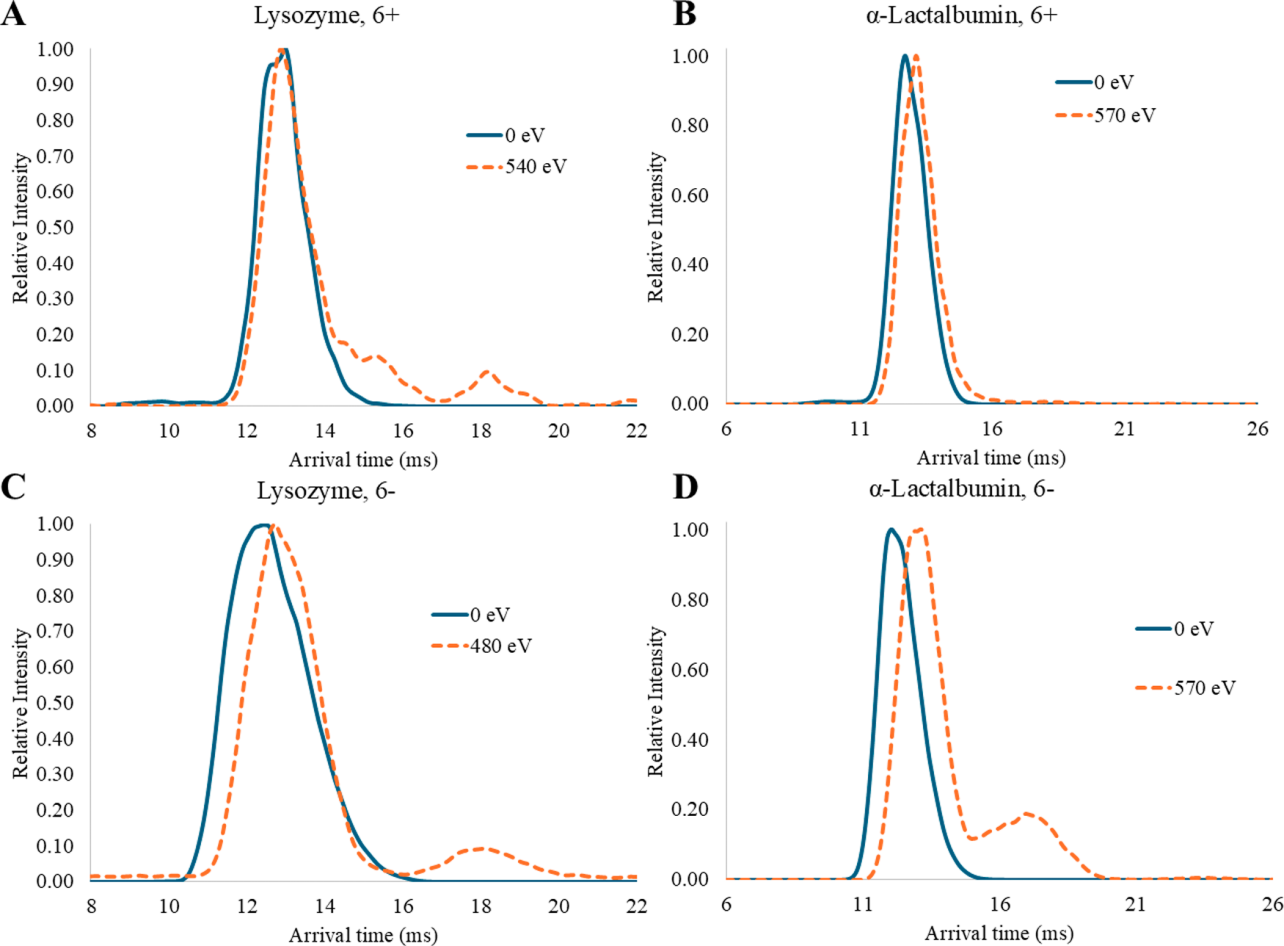
Representative mobiligrams showing arrival times for lysozyme (A, C) and α-lactalbumin (B, D) at low and high CV (blue solid and orange dashed lines, respectively) for +ESI (A, B) and −ESI (C, D). For lysozyme, different values were chosen for the high CV (*e*.*g*., 540 eV in +ESI and 480 eV in −ESI) because these were the last analyzed CV where the signal-to-noise ratio (S/N) for the 6 ± ions was greater than 3.

To characterize the stabilizing effect of disulfides, MD simulations were conducted that heat denatured 6+ ions of lysozyme and α-lactalbumin with disulfide bridges either present or removed with the resulting cysteine residues being protonated prior to heating (Figure S4). Structural changes associated with heat denaturation were characterized by taking the root-mean-square deviation (RMSD) of the heated structures relative to initial, crystal structures. Over ten trials, lysozyme ions possessed an average RMSD of (1.6 ± 0.2) nm when disulfides were present which increased to (3.5 ± 0.5) nm when disulfides were removed. Similarly, the RMSD of α-lactalbumin ions increased from (1.8 ± 0.1) nm when disulfides were present to (3.7 ± 0.3) nm when disulfides were removed, suggesting that the disulfides serve as a barrier to unfolding. This is visually represented in Figure S4C,D where simulated structures of heated lysozyme and α-lactalbumin ions with intact disulfides adopt more condensed configurations relative to those with disulfides removed. In essence, the disulfides “tie” disparate regions of protein together, which serves as a barrier to more thorough unfolding even at a maximum simulated temperature of 900 K, explaining the similarity of ^TW^CCS_N2→He_ values in +ESI versus −ESI as well as the limited percentage of ions that undergo a change in ^TW^CCS_N2→He_ upon additional collisional activation.

WGA has 16 disulfide bridges per monomeric subunit (Table 1). On average, these disulfides encompass seven amino acids each, with ∼78% the total amino acids in WGA being encompassed by at least one disulfide bridge (Figure S3). Compared to lysozyme and α-lactalbumin, the presence of disulfide bridges across small numbers of amino acids enables WGA to unfold with ΔCCS of (370 ± 60) Å^2^ and (390 ± 120) Å^2^ in +ESI and −ESI, respectively (Table 2). However, in comparison to the other lectins, GNA and con A, we hypothesize that these disulfide bridges in WGA limit the extent of unfolding, resulting in the extended, ^TW^CCS_N2→He_ values being within experimental uncertainty in both ion modes.

To summarize, lysozyme and α-lactalbumin exhibit similar gas-phase behavior in both +ESI and −ESI, with most ions retaining folded structures even under high CV. We hypothesize this resistance to unfolding is largely attributed to their four disulfide bridges, which span over 90% of their sequences and act as stabilizing constraints. MD simulations confirm that removing these disulfides significantly increases structural deviation during heat denaturation, indicating their critical role in maintaining compact configurations. Consequently, the presence of disulfide bridges explains the minimal changes in ^TW^CCS_N2→He_ and the proteins’ high stability under activation.

### Proteins Often Require More Energy to Unfold in the Polarity Matching Their Solution-Phase Charge

Hong et al. reported that avidin and β-lactoglobulin required different amounts of energy to unfold in +ESI and −ESI, resulting in different intermediate species.^29^ We hypothesize that this behavior is related to differences in a protein’s net solution- versus gas-phase charge. Therefore, using the proteins in Table 1, we quantified the CIU50 to compare the energy required to unfold proteins with varying pI.

Of the analyzed proteins that are negatively charged in solution, four of the five (monomeric ovalbumin, dimeric GNA, monomeric β-lactoglobulin, and monomeric con A) required significantly more energy to unfold in -ESI compared to +ESI (Figures 3A, S5, and S6). For example, with ovalbumin the 11+ ions have a CIU50 of (460 ± 30) eV, while the 11– ions have a CIU50 of (560 ± 30) eV. Therefore, the energy required to unfold ovalbumin in +ESI is significantly lower than in −ESI, though the ions exhibited similar folded and extended ^TW^CCS_N2→He_. This indicates that the positive-ions are less stable than the negative-ions because less energy was required to unfold the ions.

**Figure 3:**
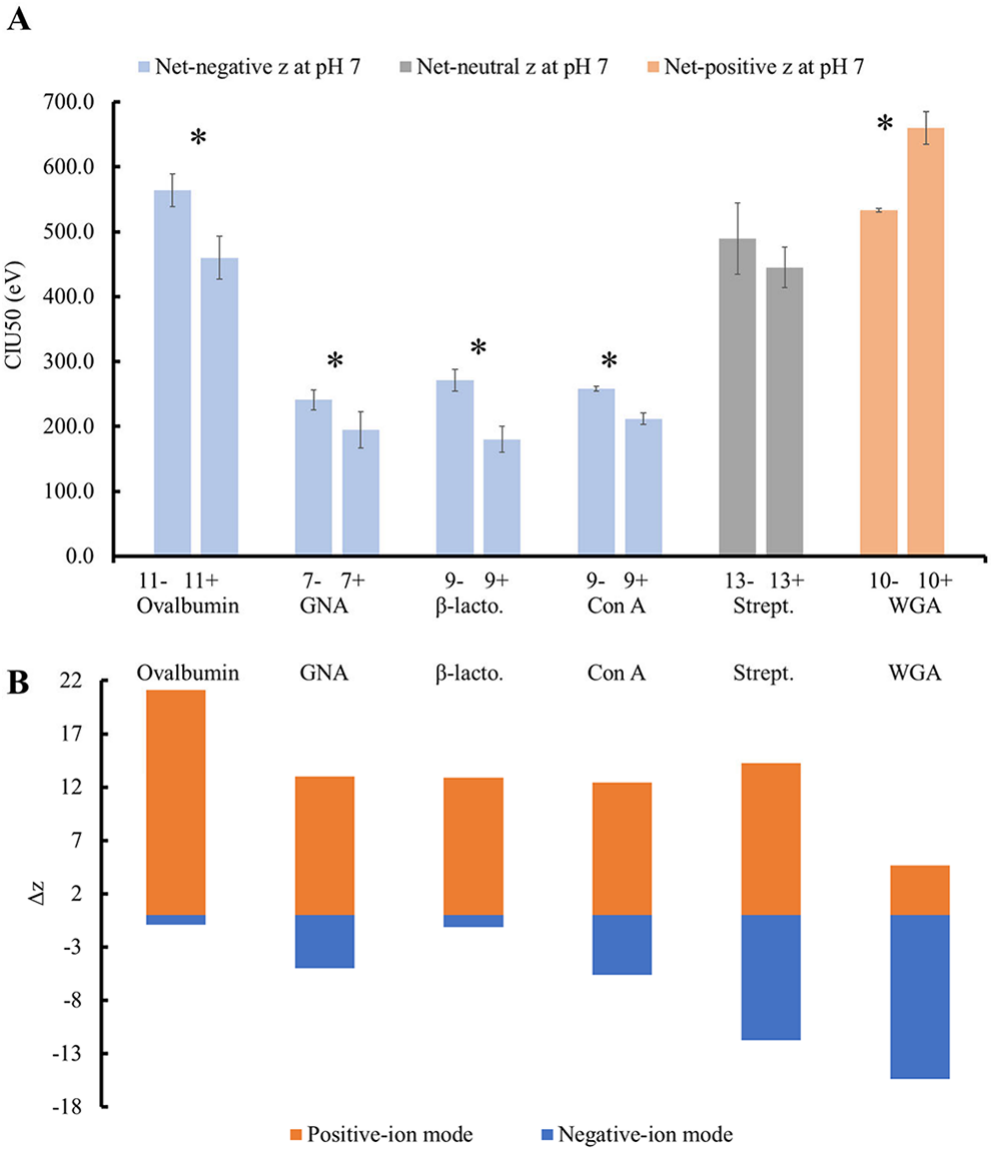
Proteins require more energy to unfold in the polarity that results in the smallest change-in-charge (Δ*z*) compared to their solution-phase charge. (A) Most proteins require more energy to unfold in one polarity. Each bar represents the average CIU50 with error bars corresponding to the standard deviation for the listed charge state. Proteins that have a net-negative, neutral, or net-positive charge in solution at pH 7 are in blue, gray, and orange, respectively. A star represents a statistical difference at the 95% CI. (B) Gas-phase proteins experience a larger Δ*z* in the polarity opposite their net-charge in solution at pH 7. The top portion (orange) represents the Δ*z* for +ESI, and the bottom portion (blue) represents the Δ*z* for −ESI. Δ*z* was determined by comparing the charge states analyzed in +ESI and −ESI (in part A) to the theoretical charge in solution at pH 7.0 using PROPKA. The following abbreviations are used: β-lactoglobulin (β-lacto.) and streptavidin (Strept.). (A and B) Proteins are listed from left to right in order of increasing theoretical charge in solution at pH 7.

Ovalbumin, GNA, β-lactoglobulin, and con A all experience a larger change-in-charge (Δ*z*) during +ESI compared to −ESI, due to the proteins having a net-negative charge in solution (Table 1 and Figure 3B). For example, ovalbumin has a predicted solution-phase charge of −10.1 at pH 7 with a pI of ∼5.19. Thus, the 11+ ions experienced a Δ*z* during ESI of +21.1, while the 11– ions experienced a Δ*z* of −0.900. This is concerning because most proteins, regardless of their net-charge in solution, are routinely analyzed in +ESI.

Tetrameric streptavidin, which has a solution-phase charge of −1.23 at pH 7 due to its pI of ∼6.71, has a near-neutral net charge. The 13 ± ions were routinely observed to have comparable folded ^TW^CCS_N2→He_ that unfold to different extended ^TW^CCS_N2→He_. The 13+ and 13- ions required similar energy, (450 ± 30 eV) and (490 ± 60 eV), respectively, to unfold (Figure 3A). For this protein, the 13+ ions experience a Δ*z* of +14.2 while the 13– ions experience a Δ*z* of −11.8 (Table 1 and Figure 3B). The streptavidin Δ*z* values are more comparable than the Δ*z* observed for ovalbumin and the other proteins with a greater net-negative charge in solution. The energy required for streptavidin to unfold in +ESI and −ESI are comparable within experimental uncertainty, suggesting that this protein does not exhibit varying gas-phase stability when analyzed in either ion mode. We hypothesize that proteins with net-neutral charge in solution, which experience similar Δ*z* during +ESI and −ESI, have comparable stability in both ion modes.

We hypothesize that proteins with a net-positive charge in solution, such as WGA and cytochrome *c*, will be more stable in +ESI, due to the smaller Δ*z*. WGA has a theoretical solution-phase charge of +3.64 due to its pI of ∼9.05. Interestingly, the positive and negative ions have folded ^TW^CCS_N2→He_ that are significantly different, but the extended ^TW^CCS_N2→He_ are comparable within experimental uncertainty. The 10+ ions have a CIU50 of (660 ± 30) eV while the 10– ions have a smaller CIU50 of (533 ± 3) eV (Figures 3A, S5 and S6). WGA requires significantly more energy to unfold in +ESI than in −ESI. During the ESI process the 10+ ions of WGA experience a smaller Δ*z* (+6.36) compared to the Δ*z* (−13.6) of the 10– ions (Table 1 and Figure 3B). In direct contrast to the proteins with a negative or neutral charge in solution, WGA requires significantly more energy to unfold in +ESI compared to −ESI, indicating an increased structural stability in +ESI.

Cytochrome *c* has a solution-phase charge of +7.40 due to its pI of ∼11.3; this gives the 6+ ions a smaller Δ*z* (−0.40) than the 6- ions (−14.40). Cytochrome *c*’s positive and negative ions have folded ^TW^CCS_N2→He_ values that were comparable within experimental uncertainty (Table 2) but have extended ^TW^CCS_N2→He_ that are not comparable within experimental uncertainty. In fact, we see that the 6- ions have two CIU50 values of (111 ± 4) eV and (150 ± 20), for the first and second unfolding events, respectively, while the 6+ ions have a single CIU50 value of (70 ± 20, Figure 4). The lower CIU50 value for the 6+ ions may initially lead one to think that the +ESI ions require less energy to unfold than the −ESI ions, but paired with the fact that the extended structure for the 6+ ions is comparable to intermediate structure of the 6– ions, we hypothesize that the 6+ ions do not unfold to the fully extended structure exhibited by the 6– ions prior to loss of ion signal (Figures 4 and S6). Taken together, this data suggests that for cytochrome *c*, which is net-positive in solution, the 6+ ions exhibit stronger gas-phase stability than the 6– ions.

**Figure 4:**
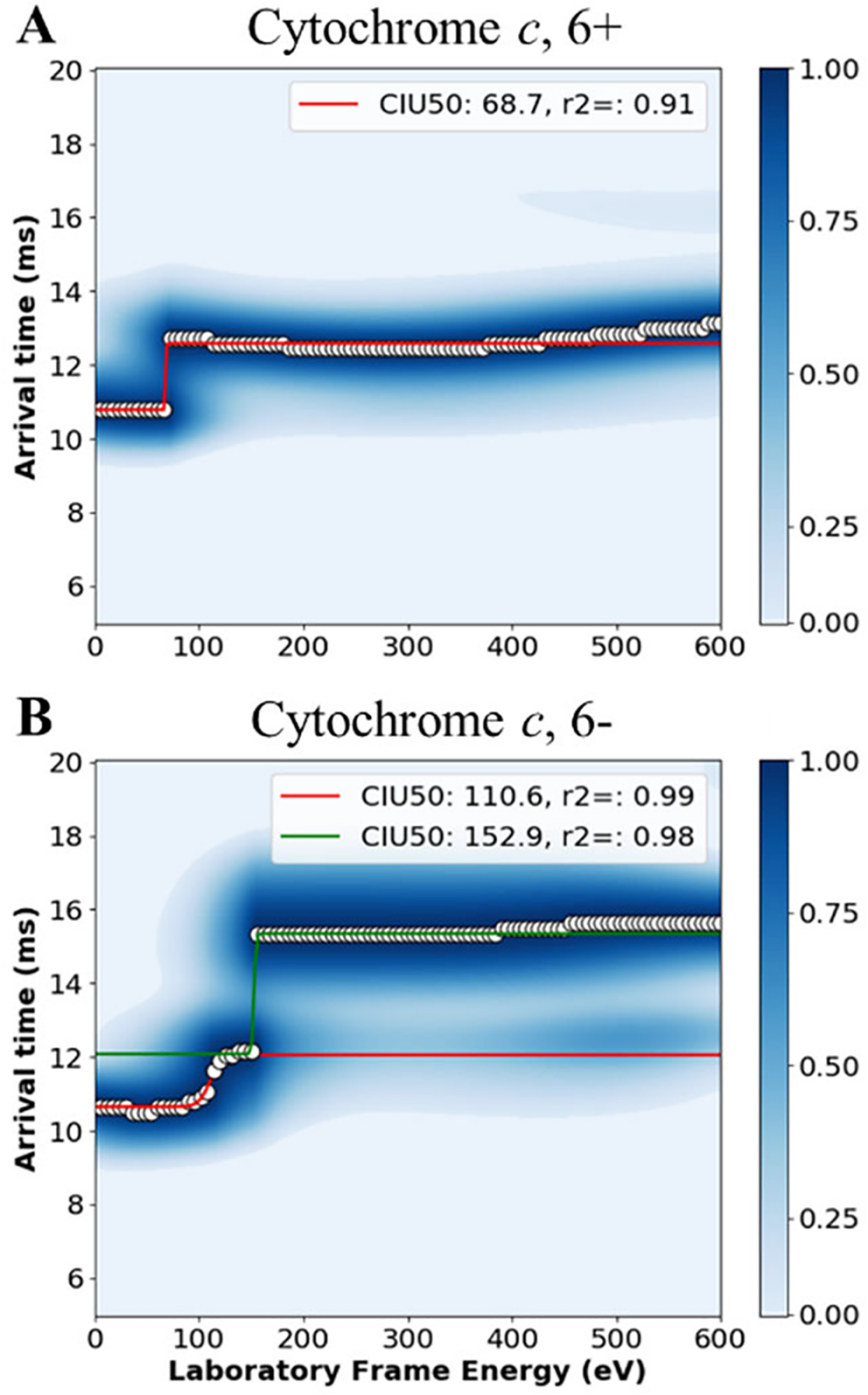
Cytochrome *c* is more stable in +ESI compared to −ESI. CIU fingerprints of cytochrome *c* for the 6+ (A) and 6– (B) ions. Cytochrome *c* has one additional unfolding event in −ESI compared to +ESI. Each fingerprint is an average of all replicates for that ion.

This data shows that there are differences in the gas-phase stability for many proteins when ionized in +ESI versus −ESI. We hypothesize that these shifts in gas-phase stability are correlated to an increase in Δ*z* relative to a protein’s solution charge. Protonation patterns in the gas-phase differ from solution, favoring neutralized rather than ionic forms of chargeable amino acids. However, upon ionization, larger swings in Δ*z* may cause more significant changes to the noncovalent interactions in gas-phase proteins relative to their “native” state. Here, proteins that were analyzed in the polarity opposite to their solution-phase charge exhibited larger Δ*z* and lower stability. Prior work showed that alterations to the electrostatic interactions at protein surfaces resulted in changes in gas-phase stability.^23^ Alternatively, streptavidin had similar Δ*z* and stability in both ion modes. Thus, we hypothesize that the intramolecular interactions for cationic and anionic streptavidin were similar in both ion modes. Furthermore, MD simulations of ESI suggest that the protonation patterns of protein ions are more similar to their biological structures when ionized in the mode that corresponds to their solution-phase charge.^16^ Thus, proteins with larger Δ*z* were expected to have more extensive restructuring of intramolecular interactions compared to the solution-phase structures.

## Conclusions

IMS-MS allows for detailed analysis of the structure and stability of native-like proteins in the gas phase. The structure of a protein is dictated by its amino acid sequence and the resulting noncovalent interactions that form the secondary, tertiary, and quaternary structures. Chargeable amino acid residues that are solvent exposed can carry charges leading to the protein having a net-positive, net-negative, or net-neutral charge in solution at physiological pH. Yet, most native IMS-MS analysis is conducted in +ESI, leading to proteins that are net-negative in solution experiencing large swings in their net-charge during the ESI process.

This work highlights that gas-phase stability of native-like proteins varies with ionization polarity. While CCS values for folded structures are generally comparable between +ESI and −ESI, unfolding energetics differ—net-negative proteins are more stable in −ESI, whereas net-positive proteins are more stable in +ESI. We hypothesize that these differences are due to changes in the intramolecular interactions within cationic and anionic proteins. Therefore, proteins analyzed in the mode opposite to their solution-phase charge may have different gas-phase structures compared to biological systems. For example, proteins that are net-negatively charged in physiological environments (pI < 6.5) are more likely to represent their biological counterparts as gas-phase anions when analyzed in −ESI compared to +ESI, enabling nMS experiments to more closely characterize the structures present in biological systems.

## Supplementary Material



## Notes

PDB numbers for the analyzed proteins, instrument parameters, ^DT^CCS_He_ calibrant values and calibration curves, tables of ^TW^CCS_N2→He_ and CIU50 values, depictions of disulfide bridges, MD simulations examining the impact of disulfide bridges, and CIU fingerprints and RMSD plots (PDF)

## †

Department of Chemistry and Physics, Indiana State University, Terre Haute, Indiana 47809, United States
