# Supplementary material for: Investigating the Effect of Isoelectric Points on the Gas-Phase Stability of Native-like Proteins Analyzed in Positive- versus Negative-Ion Mode by IMS-MS
Source: Anal Chem. 2026 Feb 4;98(6):4518–27. doi: 10.1021/acs.analchem.5c04295 (PMC12921664; doi:10.1021/acs.analchem.5c04295)
Supplement: Supplementary file 1 [file ac5c04295_si_001.pdf]

Supporting Information for:

Investigating the effect of isoelectric points on the gas-phase stability of native-like proteins analyzed in positive- versus negative-ion mode by IMS-MS

Alexis N. Edwards[1#], Madeline G. Bannon[1], Michael S. Cordes[1], Elyssia S. Gallagher[1]*

[1]Department of Chemistry and Biochemistry
Baylor University
One Bear Place #97348, Waco, TX, 76798

*Corresponding Author:
Elyssia S. Gallagher
Email: Elyssia_Gallagher@baylor.edu
Department of Chemistry and Biochemistry
Baylor University
One Bear Place #97348, Waco, TX, 76798

[#]Current Address:
Alexis N. Edwards
Department of Chemistry and Physics
Indiana State University
Terre Haute, IN 47809

TABLE OF CONTENTS

Table S1: Proteins, their source organisms, and associated PDB files.

Table S2: Instrumental parameters and voltages.

Table S3: Selected charge states for denatured calibrants and their corresponding literature drift tube CCS ($^{DT}CCS_{He}$) values.

Figure S1: Calibration curves, using denatured calibrants, for calculating $^{TW}CCS_{N2\rightarrow He}$ in +ESI and -ESI.

Table S4: Folded and extended $^{TW}CCS_{N2\rightarrow He}$ and CIU50 values for the 10 analyzed proteins at each detected charge state.

Figure S2: CIU fingerprints and RMSD plots for α-lactalbumin and lysozyme.

Figure S3: Representative depictions of disulfide bridges in the protein sequences for lysozyme, α-lactalbumin, WGA, ovalbumin, β-lactoglobulin, and GNA.

Figure S4: MD simulations show that disulfide bridges prevent significant unfolding of lysozyme and α-lactalbumin as they denature.

Figure S5: CIU fingerprints for monomeric ovalbumin, dimeric GNA, monomeric β-lactoglobulin, monomeric con A, tetrameric streptavidin, and dimeric WGA.

Figure S6: RMSD plots for monomeric ovalbumin, dimeric GNA, monomeric β-lactoglobulin, monomeric con A, tetrameric streptavidin, dimeric WGA, and cytochrome *c*.

References

Table S1: Proteins, their source organisms, and associated PDB files.

Protein	Source	PDB[a]
Ovalbumin	Chicken egg	1OVA
β-lactoglobulin	Bovine milk	1B8E
α-lactalbumin	Bovine milk	1F6R
Galanthus Nivalis agglutinin, GNA	*Galanthus nivalis*	1NIV[b,c]
Concanavalin A, con A	*Canavalia ensiformis*	1DQ6
Streptavidin	*Streptomyces avidinii*	1SWB
Ubiquitin	Bovine erythrocytes	5TR4
Wheat germ agglutinin, WGA	Wheat germ agglutinin	9WGA
Cytochrome *c*	Equine heart	1HRC
Lysozyme	Chicken egg	1DPX

[a] PROPKA v3.5.0[1-2] was used to determine the pI of each protein using their corresponding crystal structure.
[b] Indicates PDB files that were modified prior to PROPKA analysis by removal of ligands that were present.
[c] Indicates PDB files where the crystalized oligomeric state was a tetramer, which was modified to a dimer for PROPKA analysis.

Table S2: Instrumental parameters and voltages.

Parameter	Value
Trap Gas Flow Rate (mL/min)	4.00*
He Gas Flow Rate (mL/min)	150.*
IMS Gas Flow Rate (mL/min)	20.0*
Transfer Wave Velocity (m/s)	120.*
Transfer Wave Height (V)	2.00 / 7.00*
IMS Wave Velocity (m/s)	350.*
IMS Wave Height (V)	7.00* / 9.00* [a]
Sampling Cone (V)	10.0*
Source Offset (V)	10.0*
Static Offset (V)	180.*
Trap DC Entrance (V)	3.00*
Trap DC Bias (V)	20.0*
Trap DC (V)	1.00*
Trap DC Exit (V)	0.00*
IMS DC Entrance (V)	10.0*
Helium Cell DC (V)	20.0*
Helium Exit (V)	-10.0*
IMS Bias (V)	3.00*
IMS DC Exit (V)	0.00*
Transfer DC Entrance (V)	5.00*
Transfer DC Exit (V)	15.0*
Backing Pressure (mbar)	3.06 ± 0.03 [b]
Source Pressure (mbar)	$(6.45 \pm 0.06) \times 10^{-3}$ [b]
Trap Pressure (mbar)	$(2.35 \pm 0.09) \times 10^{-2}$ [b]
Helium Cell Pressure (mbar)	$(1.350 \pm 0.004) \times 10^{3}$ [b]
Transfer Pressure (mbar)	$(2.28 \pm 0.08) \times 10^{-2}$ [b]
IMS Pressure (mbar)	5.45 ± 0.01 [b]
TOF Pressure (mbar)	$(6.46 \pm 0.02) \times 10^{-7}$ [b]

* Indicates a user-specified value.
[a] An IMS wave height of 7.00 V was used for α-lactalbumin, ubiquitin, cytochrome *c*, and lysozyme. An IMS wave height of 9.00 V was used for all other proteins.
[b] Pressure readings represent the recorded average ± the standard deviation.

The electric potential at each user-controlled point on the Synapt G2-S was optimized prior to these experiments and kept consistent in both polarities to limit unintended protein

activation as the ions travelled through the instrument. The real-time electric potential at each user-controlled point was monitored as CIU experiments progressed, to ensure consistency between +ESI and -ESI, though these values are not recorded by the instrument.

Table S3: Selected charge states for denatured calibrants and their corresponding literature drift tube CCS ($^{DT}CCS_{He}$) values.[3-4]

<table>
<tr><th>Protein</th><th>z</th><th>$^{DT}CCS_{He}$ in +ESI (Å^2)</th><th>$^{DT}CCS_{He}$ in -ESI (Å^2)</th></tr>
<tr><td rowspan="17">Cytochrome c</td><td>17±</td><td>2723</td><td>n/a</td></tr>
<tr><td>16±</td><td colspan="2">2679</td></tr>
<tr><td>15±</td><td colspan="2">2579</td></tr>
<tr><td>14±</td><td colspan="2">2473</td></tr>
<tr><td>13±</td><td colspan="2">2391</td></tr>
<tr><td>12±</td><td colspan="2">2335</td></tr>
<tr><td>11±</td><td colspan="2">2303</td></tr>
<tr><td>10±</td><td colspan="2">2226</td></tr>
<tr><td>9±</td><td>2215
1964</td><td>2024*</td></tr>
<tr><td>8±</td><td>2061
1845</td><td>1702</td></tr>
<tr><td>7±</td><td>2007
1247</td><td>1620
1247</td></tr>
<tr><td>6±</td><td>1244</td><td>1244
1602</td></tr>
<tr><td>5±</td><td>n/a</td><td>1196</td></tr>
<tr><td rowspan="16">Apo-myoglobin</td><td>22±</td><td colspan="2">3815</td></tr>
<tr><td>21±</td><td colspan="2">3792</td></tr>
<tr><td>20±</td><td colspan="2">3682</td></tr>
<tr><td>19±</td><td colspan="2">3570</td></tr>
<tr><td>18±</td><td colspan="2">3489</td></tr>
<tr><td>17±</td><td colspan="2">3384</td></tr>
<tr><td>16±</td><td colspan="2">3313</td></tr>
<tr><td>15±</td><td colspan="2">3230</td></tr>
<tr><td>14±</td><td colspan="2">3143</td></tr>
<tr><td>13±</td><td colspan="2">3136</td></tr>
<tr><td>12±</td><td colspan="2">3044</td></tr>
<tr><td>11±</td><td>2942</td><td>2879*</td></tr>
<tr><td>10±</td><td>1897
2796</td><td>1897
2656*</td></tr>
<tr><td>9±</td><td>1758
2659</td><td>1758</td></tr>
<tr><td>8±</td><td>1673
2352</td><td>1673</td></tr>
<tr><td>7±</td><td>n/a</td><td>1581*</td></tr>
<tr><td rowspan="5">Ubiquitin</td><td>11±</td><td>1802</td><td>n/a</td></tr>
<tr><td>10±</td><td>1732</td><td>n/a</td></tr>
<tr><td>9±</td><td colspan="2">1649</td></tr>
<tr><td>8±</td><td colspan="2">1622</td></tr>
<tr><td>7±</td><td>1580</td><td>1317</td></tr>
</table>

	6±	1220<br>1041<br>1525	1220<br>1041
	5±	n/a	1027
*$^{DT}CCS_{He}$ of anionic species.			

Some proteins have multiple arrival times due to multiple, distinct structures being measured. When applicable, the same $^{DT}CCS_{He}$ was used for the cationic and anionic denatured calibrants.[3-4] If the arrival time varied significantly between +ESI and -ESI, due to a different structure being measured, the anionic $^{DT}CCS_{He}$ value was used for calibrating in -ESI.

Figure S1. Calibration curves, using denatured calibrants, for calculating $^{TW}CCS_{N2\rightarrow He}$ in (A, C) +ESI and (B, D) -ESI. Separate calibration curves were made for proteins that required IMS wave heights of (A, B) 7 V and (C, D) 9 V.

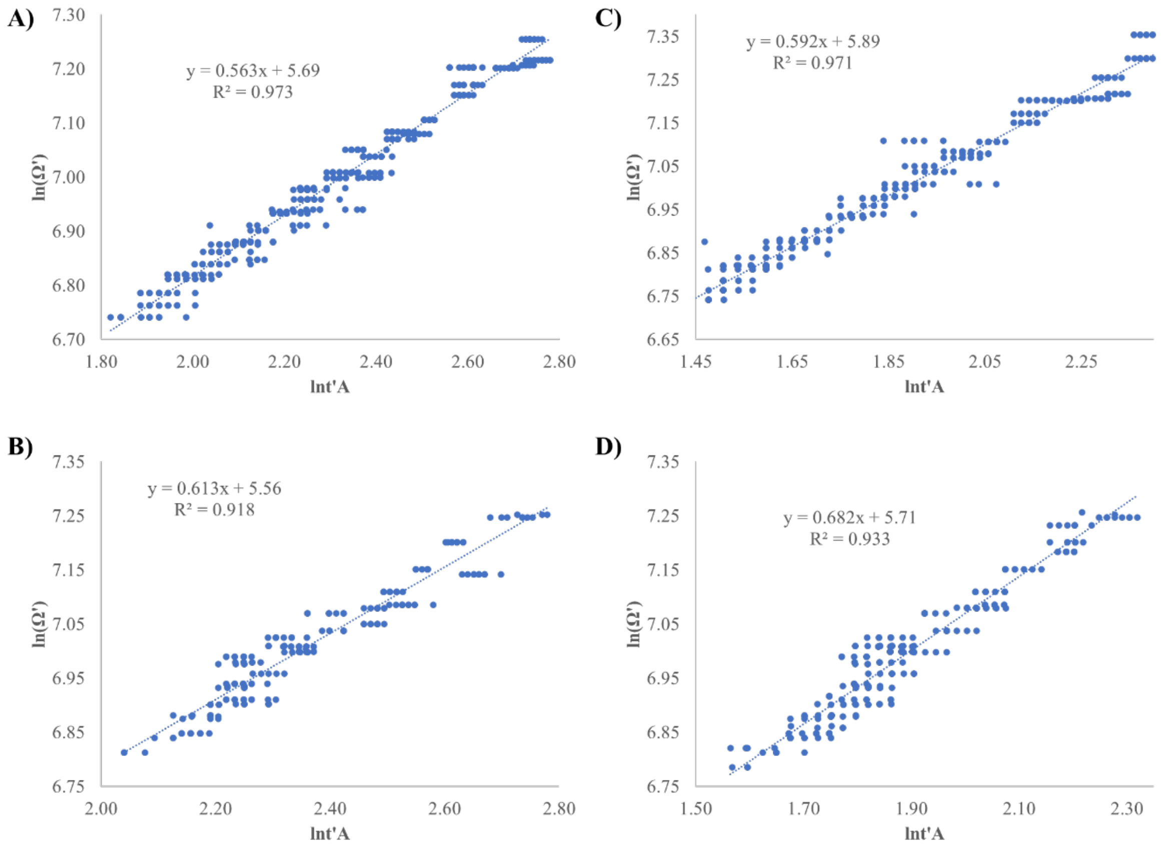

Table S4: Folded and extended $^{TW}CCS_{N2\rightarrow He}$ and CIU50 values for the 10 analyzed proteins at each detected charge state.

Protein[a]	z	Folded $^{TW}(CCS \pm CCS_{uncert})_{N2\rightarrow He}$ (Å^2)	n[b]	Extended $^{TW}(CCS \pm CCS_{uncert})_{N2\rightarrow He}$ (Å^2)	n[b]	1st CIU50 (eV)[c]	2nd CIU50 (eV)[c]
Ovalbumin	9-	3300 ± 110	8	4200 ± 150	8	790 ± 60	-
	10-	3300 ± 100	9	4200 ± 140	9	570 ± 30	-
	11-	3500 ± 110	9	4000 ± 130	9	560 ± 30	1420 ± 50
	11+	3400 ± 50	9	4040 ± 60	9	460 ± 30	1230 ± 90
	12-	3400 ± 100	9	4800 ± 160	9	550 ± 30	1270 ± 40
	12+	3380 ± 50	9	4730 ± 70	9	430 ± 30	810 ± 90
	13+	3660 ± 50	9	4560 ± 70	9	420 ± 50	650 ± 60
GNA, dimer	9-	2180 ± 60	9	2540 ± 80	9	240 ± 20	-
	9+	2100 ± 30	9	2670 ± 40	9	200 ± 30	-
β-lactoglobulin, monomer	6-	1700 ± 40	9	-		-	-
	7-	1640 ± 40	9	1880 ± 50	9	290 ± 20	-
	7+	1640 ± 40	9	1960 ± 30	9	170 ± 20	-
	8+	1700 ± 20	9	2160 ± 30	9	170 ± 20	400 ± 20
	9+	1780 ± 30	9	2080 ± 30	9	-	-
β-lactoglobulin, dimer	9-	2820 ± 70	8	3010 ± 80	8	430 ± 30	590 ± 20
	10-	3040 ± 80	9	3550 ± 100	9	480 ± 10	-
	11+	3060 ± 40	9	3630 ± 50<br>3870 ± 50	9<br>9	390 ± 30	590 ± 90
	12+	3020 ± 40	9	3540 ± 50<br>3870 ± 50	9<br>9	360 ± 30	780 ± 30
	13+	3010 ± 30	9	3600 ± 60<br>4070 ± 50	9<br>9	330 ± 30	930 ± 40
α-lactalbumin	5-	1410 ± 50	9	-	-	410 ± 40[c]	-
	6-	1380 ± 40	8	-	-	470 ± 30[c]	-
	6+	1410 ± 20	8	-	-	440 ± 20[c]	-
	7-	1410 ± 40	9	1700 ± 60	8	130 ± 10	-
	7+	1430 ± 20	9	1710 ± 20	9	130 ± 20	-
	8+	1490 ± 20	9	1790 ± 20	7	80 ± 20	-

Con A, monomer	9-<br>9+	2150 ± 70<br>2090 ± 40	9<br>9	2840 ± 70<br>2880 ± 50	9<br>9	258 ± 4<br>210 ± 20	-<br>390 ± 20
Streptavidin	12-	4220 ± 140	9	4750 ± 110	9	520 ± 50	-
	13-<br>13+	3900 ± 120<br>3970 ± 60	9<br>9	4700 ± 150<br>4960 ± 70	9<br>9	490 ± 60<br>450 ± 30	-<br>-
	14-<br>14+	3800 ± 110<br>3810 ± 60	9<br>9	4700 ± 150<br>4970 ± 70	9<br>9	460 ± 60<br>420 ± 30	-<br>-
	15+	3650 ± 60	9	5100 ± 90	9	410 ± 60	-
Ubiquitin	4-<br>4+	970 ± 30<br>990 ± 10	9<br>8	-<br>-	-<br>-	-<br>-	-<br>-
	5-<br>5+	960 ± 30<br>990 ± 20	9<br>9	1020 ± 40<br>1070 ± 20	8<br>9	90 ± 20<br>60 ± 20	-<br>-
	6+	1010 ± 10	9	1380 ± 20	9	70 ± 10	-
WGA	9-<br>9+	2620 ± 80<br>2600 ± 40	9<br>9	-<br>-	-<br>-	-<br>-	-<br>-
	10-<br>10+	2680 ± 80<br>2560 ± 50	9<br>9	3070 ± 90<br>2920 ± 40	9<br>9	533 ± 3<br>660 ± 30	-<br>-
Cytochrome *c*	5-<br>5+	1260 ± 40<br>1280 ± 20	9<br>9	-<br>-	-<br>-	-<br>-	-<br>-
	6-<br>6+	1240 ± 40<br>1260 ± 20	9<br>9	1570 ± 50<br>1380 ± 20	9<br>9	111 ± 4<br>70 ± 20	150 ± 20<br>-
	7+	1350 ± 30	9	1750 ± 20	9	60 ± 10	-
Lysozyme	5-	1450 ± 50	9	1750 ± 60	9	-	-
	6-<br>6+	1390 ± 40<br>1420 ± 20	9<br>9	1720 ± 60<br>1710 ± 20	9<br>9	-<br>-	-<br>-
	7-<br>7+	1720 ± 60<br>1710 ± 20	9<br>9	2130 ± 70<br>2110 ± 30	9<br>9	-<br>520 ± 20	-<br>-

[a] Proteins are listed in order of increasing theoretical charge at pH 7.
[b] n represents the number of replicates.
[c] CIU50 values were calculated in CIUSuite2 for each experimental replicate and then averaged to present an average ± standard deviation. See the *Methods* in the paper for additional details.

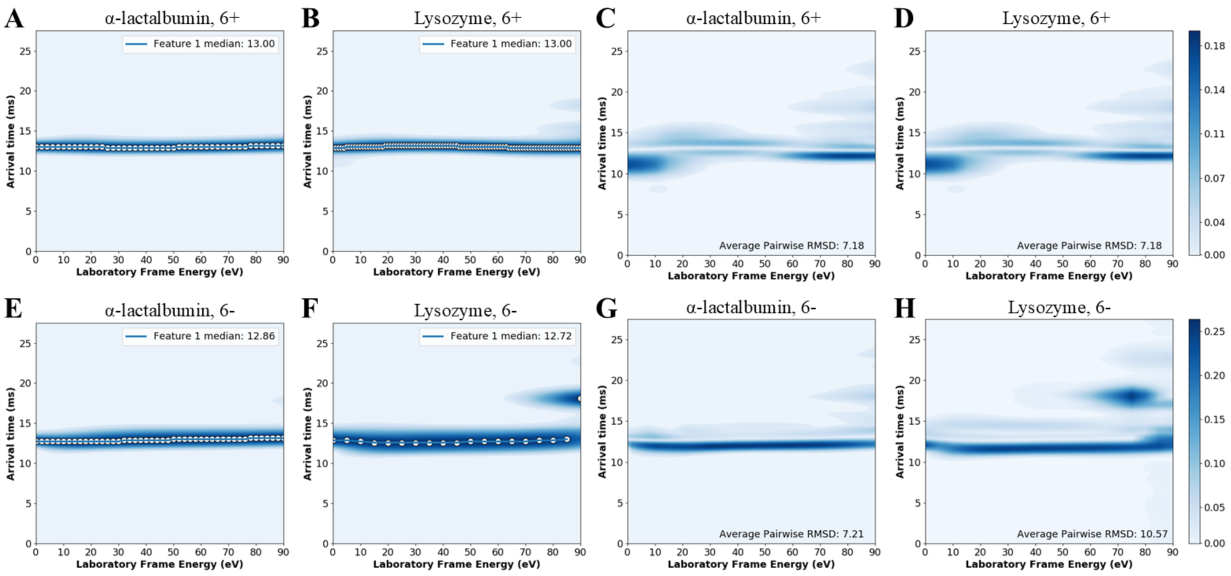

Figure S2: CIU fingerprints (A, B, E, F) and RMSD plots (C, D, G, H) for α-lactalbumin (A, C, E, G) and lysozyme (B, D, F, H). CIU fingerprints were prepared in CIUSuite2 using the "average" function; therefore, each image is based on all replicate analyses (n is a minimum of eight replicates). RMSD plots (C, D, G, H) shows the RMSD calculated based on the replicate analyses that were used to generate the CIU fingerprints (A, B, E, F). Dark blue traces on these heatmaps indicate regions where individual replicates differed from one another.

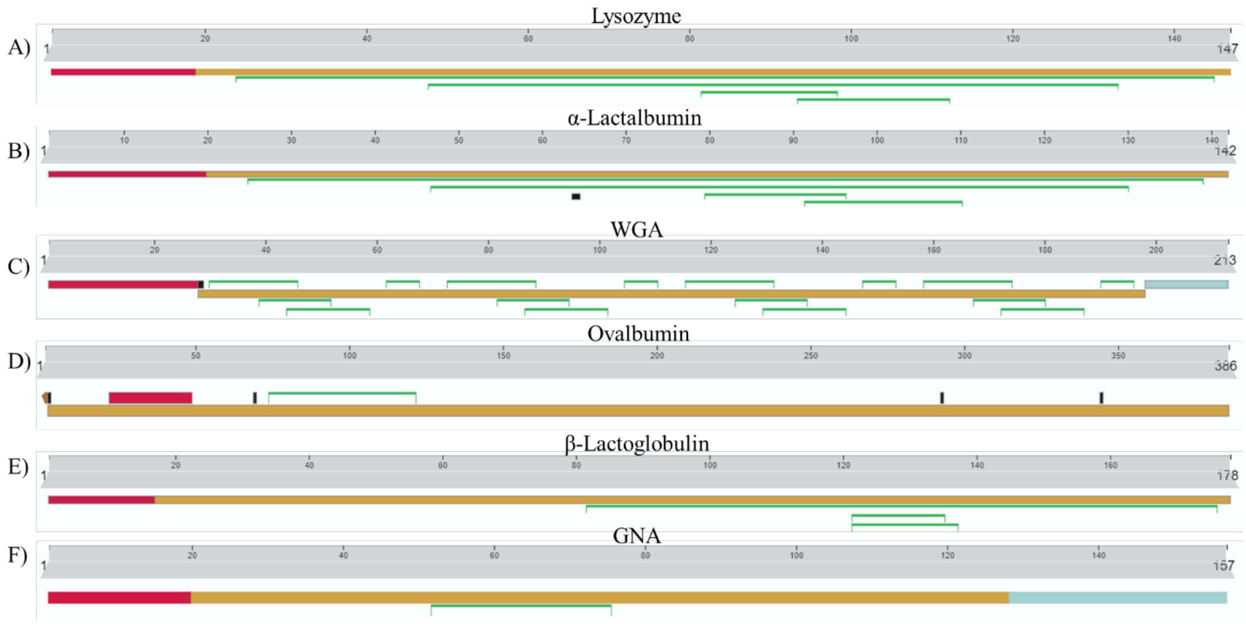

Figure S3: Representative depictions of disulfide bridges in protein sequences, depicted as green brackets, for (A) lysozyme, (B) α-lactalbumin, (C) WGA, (D) ovalbumin, (E) β-lactoglobulin, and (F) GNA. Gray bars with imbedded numbers indicate the amino acid sequence. Amino acids that have a red bar below them indicate the prepeptide (signal sequence), while those that have a blue bar indicate the propeptide, both of which are cleaved prior to full maturation of the protein. Figures were saved from UniProt, which has a Creative Commons Attribution 4.0 International (CC BY 4.0) License. https://creativecommons.org/licenses/by/4.0/

Lysozyme and α-lactalbumin both have four disulfide bridges that encompass a large percentage of the amino acid sequences (A and B, respectively). These four disulfide bridges encompass ~94% and ~93% of the amino acids in lysozyme and α-lactalbumin, respectively. We hypothesize that lysozyme and α-lactalbumin have less opportunity to unfold in CIU experiments due to a higher percentage of their amino acid sequence, and in turn their secondary and tertiary structures, being stabilized by the four disulfide bridges present in each protein.

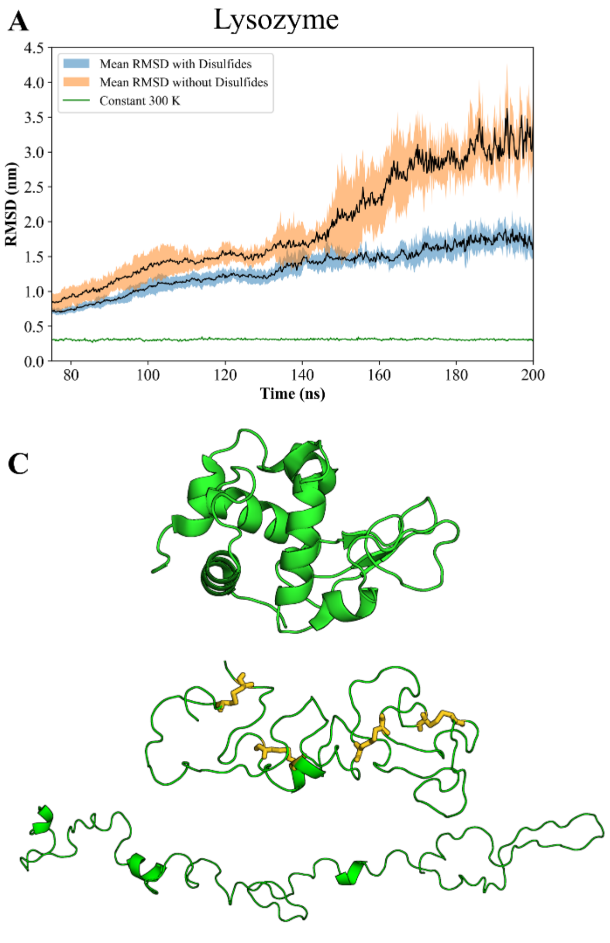

Figure S4: MD simulations show that disulfide bridges prevent significant unfolding of (A, C) lysozyme and (B, D) α-lactalbumin as they denature.

Molecular Dynamics Simulations. Considering charge states from experimental mass spectra, a 6+ charge state was assigned to both lysozyme and α-lactalbumin using the charge placement algorithm from Collidoscope[5] to determine amino-acid protonation states. Gaseous, ionic proteins were energy minimized and equilibrated in the gas phase prior to being heated from 300 K to 900 K over 200 ns. The level of denaturation with increasing temperature was assessed with root mean square deviation (RMSD) values relative to initial, crystal structures. To determine the level of stability afforded by the presence of disulfides. Disulfide bridges were

either left intact, or manually removed within GROMACS[6] by protonation of the Cys residues prior to starting the MD simulation. Ten replicates of each protein with and without disulfide bridges were conducted.

Discussion of Figure S4. Levels of denaturation for lysozyme (A) and α-lactalbumin (B) were assessed with root mean square deviation (RMSD) values relative to initial, crystal structures. Specifically, we analyzed the proteins at a constant temperature of 300 K (green trace), and increasing temperature over 200 ns for both proteins with and without disulfide bridges (blue and orange traces, respectively). For both lysozyme (C) and α-lactalbumin (D), the proteins retain more condensed structures as the proteins are thermally denatured if disulfide bridges are retained. This is observed in the representative structures for lysozyme (C) and α-lactalbumin (D), showing the folded structures (top), heat denatured structures with intact disulfide bridges (colored in yellow) (middle), and heat denatured structures lacking disulfide bridges (bottom).

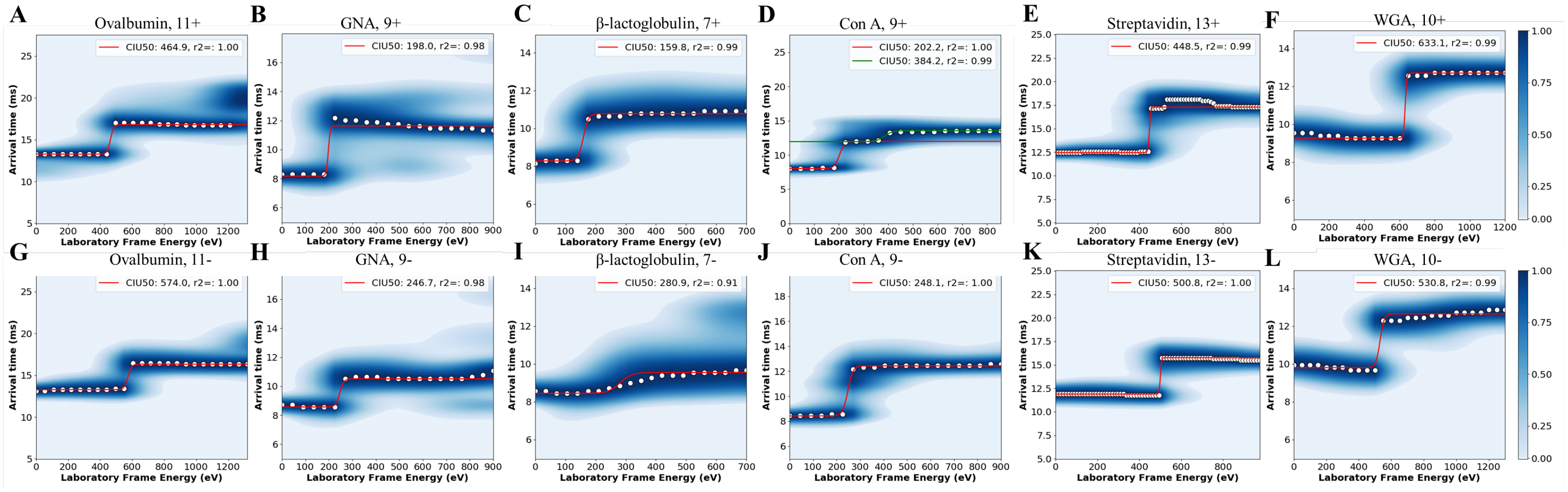

Figure S5: Many proteins require more energy to unfold in the polarity that matches their solution-phase charge at pH 7. CIU fingerprints of proteins with net-negative, net-neutral, and net-positive solution-phase charge at pH 7. CIU fingerprints for overlapping charge states of (A, G) monomeric ovalbumin, (B, H) dimeric GNA, (C, I) monomeric β-lactoglobulin, (D, J) monomeric con A, (E, K) tetrameric streptavidin, and (F, L) dimeric WGA. CIU fingerprints were prepared in CIUSuite2 using the "average" function; therefore, each image is based on all replicate analyses (n is a minimum of eight replicates).

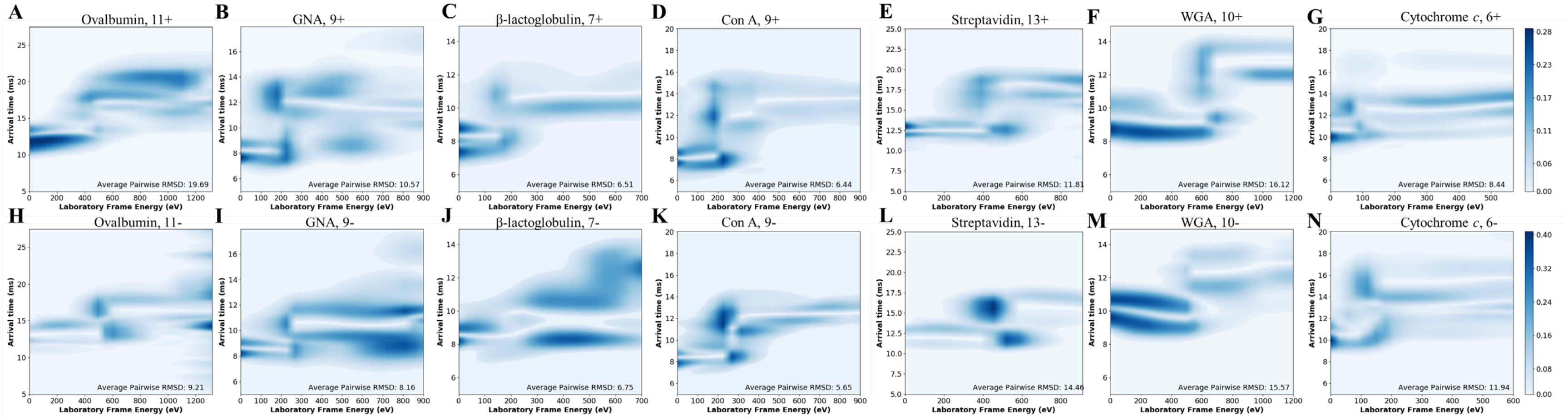

Figure S6: RMSD plots for (A, H) monomeric ovalbumin, (B, I) dimeric GNA, (C, J) monomeric β-lactoglobulin, (D, K) monomeric con A, (E, L) tetrameric streptavidin, (F, M) dimeric WGA, and (G, N) cytochrome *c*. Each plot shows the RMSD calculated based on the replicate analyses that were used to generate the CIU fingerprints in Figure S5. Dark blue traces on these heatmaps indicate regions where individual replicates differed from one another.

References

1. Bas, D. C.; Rogers, D. M.; Jensen, J. H., Very fast prediction and rationalization of pKa values for protein-ligand complexes. *Proteins* **2008,** *73* (3), 765-83.
2. Olsson, M. H.; Sondergaard, C. R.; Rostkowski, M.; Jensen, J. H., PROPKA3: Consistent Treatment of Internal and Surface Residues in Empirical pKa Predictions. *J Chem Theory Comput* **2011,** *7* (2), 525-37.
3. Shelimov, K. B.; Clemmer, D. E.; Hudgins, R. R.; Jarrold, M. F., Protein Structure in Vacuo: Gas-Phase Conformations of BPTI and Cytochrome c. *J Am Chem Soc* **1997,** *119* (9), 2240-2248.
4. Valentine, S. J.; Counterman, A. E.; Clemmer, D. E., Conformer-dependent proton-transfer reactions of ubiquitin ions. *J Am Soc Mass Spectr* **1997,** *8* (9), 954-961.
5. Ewing, S. A.; Donor, M. T.; Wilson, J. W.; Prell, J. S., Collidoscope: An Improved Tool for Computing Collisional Cross-Sections with the Trajectory Method. *J Am Soc Mass Spectrom* **2017,** *28* (4), 587-596.
6. Van Der Spoel, D.; Lindahl, E.; Hess, B.*, et al.*, GROMACS: Fast, flexible, and free. *J Comput Chem* **2005,** *26* (16), 1701-1718.
